# Wearable Sensor-Based Monitoring of Environmental Exposures and the Associated Health Effects: A Review

**DOI:** 10.3390/bios12121131

**Published:** 2022-12-06

**Authors:** Xueer Lin, Jiaying Luo, Minyan Liao, Yalan Su, Mo Lv, Qing Li, Shenglan Xiao, Jianbang Xiang

**Affiliations:** 1 School of Public Health (Shenzhen), Sun Yat-sen University, Shenzhen 518107, China; 2 College of Medicine and Biological Information Engineering, Northeastern University, Shenyang 110819, China; 3 School of Public Health (Shenzhen), Shenzhen Campus of Sun Yat-sen University, Shenzhen 518107, China; 4 State Environmental Protection Key Laboratory of Sources and Control of Air Pollution Complex, Beijing 100084, China

**Keywords:** sensor, wearable, portable, environmental monitoring, health effect, human subject study

## Abstract

Recent advances in sensor technology have facilitated the development and use of personalized sensors in monitoring environmental factors and the associated health effects. No studies have reviewed the research advancement in examining population-based health responses to environmental exposure via portable sensors/instruments. This study aims to review studies that use portable sensors to measure environmental factors and health responses while exploring the environmental effects on health. With a thorough literature review using two major English databases (Web of Science and PubMed), 24 eligible studies were included and analyzed out of 16,751 total records. The 24 studies include 5 on physical factors, 19 on chemical factors, and none on biological factors. The results show that particles were the most considered environmental factor among all of the physical, chemical, and biological factors, followed by total volatile organic compounds and carbon monoxide. Heart rate and heart rate variability were the most considered health indicators among all cardiopulmonary outcomes, followed by respiratory function. The studies mostly had a sample size of fewer than 100 participants and a study period of less than a week due to the challenges in accessing low-cost, small, and light wearable sensors. This review guides future sensor-based environmental health studies on project design and sensor selection.

## 1. Introduction

Excessive environmental exposure is a major risk factor for the worldwide burden of diseases nowadays. The Institute for Health and Evaluation (IHME) estimates that approximately 12.4 million people died in 2019 due to living or working in unhealthy environments, accounting for 20% of all global deaths [1,2]. Numerous studies have documented that exposure to environmental factors (e.g., air quality, temperature, noise) is significantly related to various human diseases and premature deaths. For instance, air pollution is associated with morbidity and mortality due to cardiovascular diseases [3], lung cancers [3], diabetes [4], Alzheimer’s diseases [5,6], depression [7], and anxiety [8]. Meanwhile, noise exposure can damage the human cardiovascular system, autonomic nervous system, endocrine system, and neurocognitive functioning [9]. Extreme environmental temperatures significantly increase the risks of cardiovascular diseases [10], neurodegenerative diseases [11], depression [12], and anxiety [13]. In addition, illumination and light can impact human cognitive functions [14].

It is often required to measure environmental exposure and health responses in environmental health-related studies. Traditionally, environmental exposure levels are typically measured using stationary monitoring stations or sampling devices placed at fixed locations in population-based studies [3,15,16,17,18,19]. For example, in a study assessing the relationship between long-term exposure to fine particles (PM_2.5_) and premature mortality, the PM_2.5_ exposure levels were obtained from public monitoring networks [3]. In another study examining the cardiorespiratory effects of various filters in building ventilation systems, the ozone exposure levels were measured using a fixed-site instrument (2B Tech Model 205) combined with time-activity questionnaires [19]. As for health response measurements, the common ways include supervised or unsupervised questionnaires [20,21,22], routine clinical visits in medical facilities [23], and portable medical devices operated by non-medical persons [19]. All these traditional means of measuring environmental exposure and health responses require either high-end instruments or operation by experienced persons, limiting the application of such measures on a large scale. In addition, with limited spatiotemporal resolutions, such measures make it challenging to measure personal environmental exposure and health responses continuously. 

In recent years, rapid advances in sensor technology have facilitated the development and use of personalized sensors [24,25]. A report predicts that the global smart sensor market will exceed USD 208 billion by 2031 [26]. As many sensors are relatively low-cost and portable and can provide data with high spatiotemporal resolutions, they have been used in many environmental health-related studies, especially in individual-monitoring aspects. For instance, Runkle et al. used wearable sensors to continuously measure individual experienced temperature and physiologic heat strain response among grounds maintenance workers [27]. Sisto et al. used portable sensors to evaluate the hearing loss of fiberglass-manufacturing workers due to noise exposure [28]. Tang et al. used mobile sensors to investigate the effects of personal exposure to particle-bound polycyclic aromatic hydrocarbons on the heart rate variability of healthy elderly [29]. 

There have been several reviews on the progress of portable sensor development and application in environmental monitoring [25,30,31,32,33,34,35,36]. For example, Willner and Vikesland reviewed nanosensor design and application in detecting environmental pesticides, heavy metals, and pathogens. Meanwhile, another review by Ullo and Sinha [33] focused on advances in intelligent environment monitoring systems using portable sensors and the Internet of Things. Additionally, Morawska et al. systematically reviewed the applications of low-cost sensing technologies for air quality monitoring and exposure assessment in large projects, with attention to sensor validation, deployment, and data access [31]. On the other hand, there are also reviews on health sensors [37,38,39,40]. Generally, the previous reviews mainly focus on developing and applying either environmental or health sensors. For instance, Meng et al. reviewed wearable pressure sensors for human pulse wave monitoring, focusing on the transduction mechanism, microengineering structures, and related applications in pulse wave monitoring and cardiovascular condition assessment [37]. Oh et al. reviewed health monitoring sensors in electronic skin, focusing on sensing mechanisms and material [38]. To our knowledge, no studies have reviewed the research advancement in examining population-based health responses to environmental exposure via portable/wearable sensors. Herein, we refer to the studies measuring both exposure and health indicators with portable/wearable sensors rather than those with sensing measurements in only one aspect. Sensors applied in both the environment and health enable a detailed time–location–exposure–health picture for each individual and a deep analysis of how environmental exposure impacts human health indicators dynamically. 

Given the gaps in the existing literature, this study aims to review studies that use portable sensors to measure both environmental factors and health responses while exploring environmental effects on health. As such, the present review and analysis guide future sensor-based environmental health studies on project design and sensor selection.

## 2. Methods

### 2.1. Data Source and Search Strategy

We searched articles published from 1 January 1990 to 31 March 2022 in two major English databases (Web of Science and PubMed). Using the Boolean phrase and wildcard character, we tried a variety of search strategies. Firstly, we focused on the “health effects” of general “environmental factors/exposure” using “wearable/mobile/portable” sensors. Secondly, we further searched the articles on a specific physical, chemical, or biological environmental factor (i.e., noise, heat, temperature, humidity, radiation, electromagnetic, UV, ozone, nitrogen oxide, ammonia, chemical, organic, inorganic, formaldehyde, volatile organic compound (VOC), particulate matter (PM), virus, bacteria, fungi). All the terms were searched in topics that included titles, abstracts, and keywords. Thirdly, the references of the included papers and other relevant papers of the lead authors that met the eligibility criteria were also reviewed. The search strategy is shown in Table S1 (Supplementary Materials). EndNote (Version X8) was used to manage the citations.

### 2.2. Eligibility Criteria

The eligibility criteria were as follows: (1) language was limited to English; (2) article types were limited to research articles; (3) both environmental exposure and health responses were measured by portable sensors/instruments rather than stationary devices or questionnaires. Meanwhile, articles lacking available full texts were excluded. 

With all the search results determined, as shown in Table S1, one reviewer performed the first screening of titles and abstracts based on the above eligibility criteria. Afterward, another reviewer examined the full text and re-screened the selected literature. Lastly, all reviewers checked the screened full text to validate quality. Disagreements were resolved by consensus after discussion.

### 2.3. Study Inclusion

**Figure 1:**
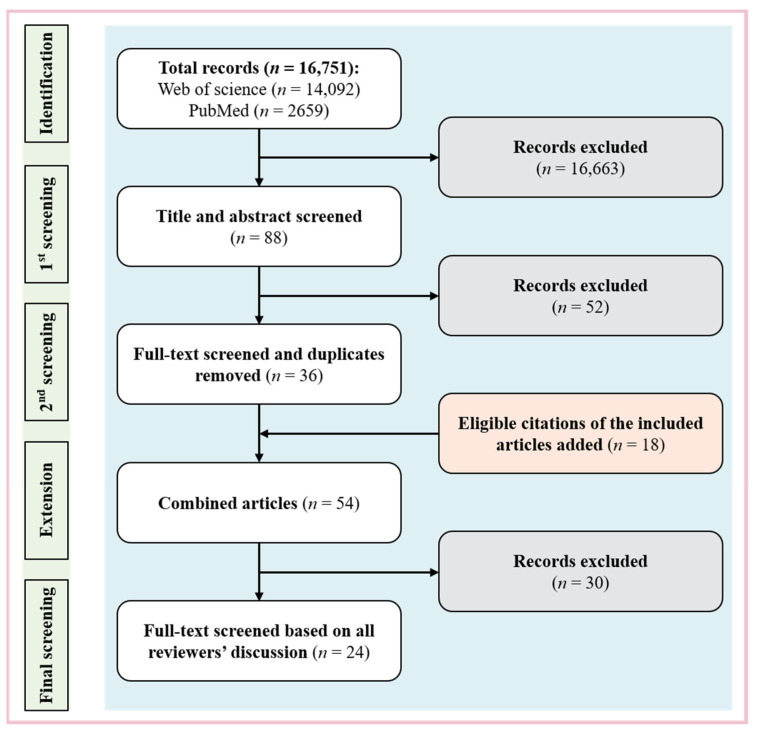
Flow chart of study inclusion.

The flow chart of study inclusion is shown in Figure 1. With the above search strategy, 16,751 records were identified in the initial search, including 14,092 from the Web of Science and 2659 from PubMed. After the 1st screening based on titles and abstracts, 88 records remained. With full texts screened by another reviewer, 36 records remained after ineligible, and duplicated records were removed. Furthermore, 18 articles were identified based on the citations of the 36 records, adding up to 54. Thirty records were further excluded after all the reviewers screened the full texts and discussed their eligibility. Finally, 24 studies were included in our analysis.

### 2.4. Analysis Strategy

The 24 included studies were summarized based on environmental factors. Specifically, the studies were separated into three major categories, i.e., physical factors, chemical factors, and biological factors. Physical factors include noise, temperature, humidity, and radiation. Chemical factors include inorganic gaseous pollutants, organic gaseous pollutants, particles, and heavy metals. In contrast, biological factors include viruses, bacteria, and fungi. Eligible studies for each subgroup were summarized in a table, with information on specific environmental factors, health indicators, sensor models, location, period of analysis, and population. Furthermore, challenges and opportunities regarding the wearable sensor-based environmental health studies were discussed. In the review, we focused on introducing the methods, particularly about sensors, of the reviewed studies rather than the results or conclusions. As such, we aim to facilitate future studies on how to design projects, especially on how to choose reliable, portable, and low-cost sensors.

## 3. Results

### 3.1. Overview

**Figure 2:**
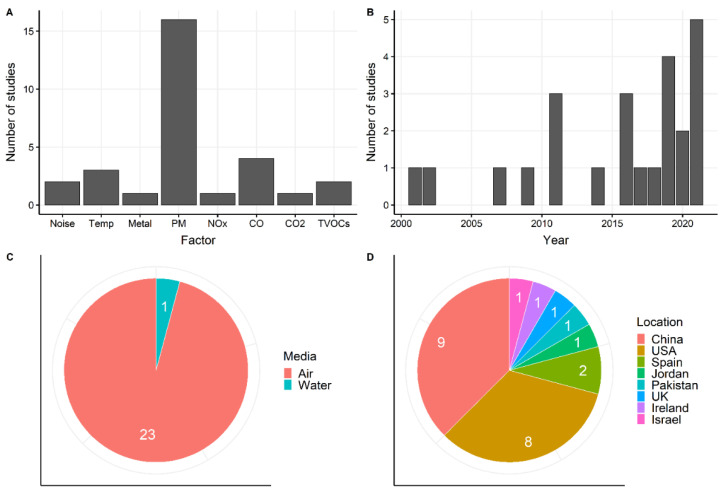
Summary of the included studies by: (**A**) environmental factors, (**B**) published year, (**C**) environmental media, and (**D**) location of field tests. Abbreviations: Temp = temperature; PM = particulate matter.

The 24 included studies are summarized in Figure 2 and Table S2, including 5 studies on physical environmental factors and 20 on chemical factors (one study involved both factors). No eligible studies on biological factors were found. Among the five studies on physical environmental factors, two examined noise, and three examined temperature. No eligible studies were found for electromagnetic, UV, visible light, or infrared radiation. As for the studies on chemical factors, 16 examined PM, six examined inorganic gaseous pollutants, two examined organic gaseous pollutants (total volatile organic compounds (TVOCs)), and one examined a heavy metal—arsenic. In addition, no eligible studies were found for ozone, sulfate oxides, ammonia, radon, or a specific VOC. Regarding environmental media, 23 studies focused on the atmospheric environment, and only one focused on the water environment. In contrast, no studies were found for the soil environment. Twenty studies were published in the past ten years, while thirteen were published in the past five years, indicating an emerging and growing research interest. The studies were primarily conducted in China (nine papers) and the USA (eight papers), with the others in some Asian and European countries. Additionally, the typical sensors and the wearable positions in the included studies are illustrated in Figure S1. 

### 3.2. Physical Factors

In this section, we found two eligible studies on noise and three on non-optimal temperature, as summarized in Table 1.

#### 3.2.1. Noise

Noise is a prominent feature of the environment, mainly including the noise from industry, construction, and traffic. Based on the IHME estimates, approximately 17.4% of hearing loss worldwide in 2019 was attributed to occupational noise exposure, corresponding to about 7 million disability-adjusted life years (DALYs) [1]. In addition, a WHO report shows that environmental noise (mainly traffic noise in the analysis) accounted for at least one million healthy life years per year in Western Europe, including 61,000 years of ischemic heart disease, 45,000 years of the cognitive impairment of children, 903,000 years from sleep disturbance, 22,000 years from tinnitus, and 587,000 years from annoyance [45]. 

We found two studies examining the health effects of noise exposure using portable sensors, as summarized in Table 1. Both studies used portable sound meters [41,42], which contain a flexible membrane that moves slightly in sound waves and converts membrane movement into an electrical signal. As for the health measurement, one study measured one-time blood pressure (BP) with a portable BP monitor operated by a technician, whereas the other measured heart rate (HR) and BP continuously for eight hours with a Holter monitor. In addition, the former focused on 191 male workers in an industrial noise exposure scenario, whereas the latter focused on 28 healthy adults in a traffic noise exposure scenario. In addition, the continuous HRV measurement enabled a high-resolution data analysis at a 5 min scale. However, the health measurements were mainly on a few cardiovascular indicators with limited portability and operability. In contrast, the investigations on hearing loss were mainly conducted via questionnaires or clinical visits, rather than portable sensors [46,47]. One study found that exposure to a high noise level was associated with elevated blood pressure [41], whereas the other study mainly reported the health effects of air pollution, as discussed below.

#### 3.2.2. Temperature

Extreme temperatures have increasingly occurred in recent years due to global warming. Based on the IHME estimates, non-optimal temperatures led to about 2 million deaths and 37.6 million DALYs worldwide in 2019, mainly from cardiovascular diseases, chronic respiratory diseases, respiratory infections, tuberculosis, diabetes, and kidney diseases [1]. Another analysis showed that the increased frequency and magnitude of heat waves affected over 125 million adults during 2000–2016, resulting in a 5.3% decrease in global outdoor manual labor productivity [48]. Non-optimal temperatures can impact human health in many aspects. Particularly, heat exposure can cause dizziness, weakness, fatigue, cramps, and fainting, and in the case of heat stroke, it can even lead to multi-organ failure, coma, and death. Meanwhile, cold exposure increases the risk of cardiovascular and respiratory disease [49].

We found three studies examining the health effects of non-optimal temperature exposure using portable sensors, as summarized in Table 1. One of the studies assessed measured the 48 h personal ambient temperature, body temperature, heart rate, and activity level of 42 elderly residents in Maryland during heat episodes, without specifying the makes and models of sensors [44]. The other two studies were from the same group, which used the same small-size button-type sensors to measure temperature and used smartwatches to measure heart rate and GPS coordinates [27,43,44]. The GPS information facilitated the distinction between outdoor and indoor personal experienced temperatures. While both targeted the outdoor occupational population, one focused on heat exposure and the other on cold exposure. In total, 35 and 54 subjects were continuously monitored for five consecutive days, respectively. One study revealed that the association between increasing temperature and individuals’ heat strain was nonlinear and exhibited a U-shaped relationship [27], whereas the other studies showed that the ground station temperature might not well represent personal sensing or body temperature.

### 3.3. Chemical Factors

#### 3.3.1. Gaseous Pollutants

In this section, we found one eligible study on nitrogen oxides (NO_x_), four on carbon monoxide (CO), one on carbon dioxide (CO_2_), and two on total VOCs (TVOCs). The details are summarized in Table 2.

- NOx.

NO_x_ is one of the most common environmental pollutants, mainly from human activities related to the combustion of fossil fuels. NO_x_ is associated with respiratory diseases, especially asthma, often resulting in many respiratory symptoms (coughing, wheezing, or difficulty breathing) [55,56]. Matt et al. studied the short-term effects of traffic-related air pollution (including NO_x_) and physical activity on the respiratory function of participants using portable sensors [50]. They focused on 30 healthy adults in Spain, each going through four 2 h traffic-related exposure scenarios during 2013–2014. A nitric oxide monitor was used to measure NOx continuously_,_ and a portable spirometer was used to assess the respiratory function three times.

- Carbon monoxide (CO) and carbon dioxide (CO_2_)

Increased CO_2_ emissions aggravate global climate change and pose a potential cardiopulmonary health risk to humans. Recent studies have shown significant linear physiological changes in circulatory, cardiovascular, and autonomic systems due to excess CO_2_ exposure [57]. In addition, CO_2_ is a significant factor associated with symptoms of sick building syndrome (SBS) in office workers [58]. On the other hand, CO, one of the five pollutants included in the Air Quality Index [59], can cause adverse reactions in the cardiovascular system, including angina pectoris at moderate exposure concentrations and myocardial infarction at high exposure concentrations [60,61]. In addition, CO can lower the partial pressure of oxygen in the blood and significantly increase the risk of heart-related complications after surgery [62]. 

All four studies on CO used electrochemical sensors to continuously measure CO levels, among which two used TSI Q-Trak [29,54], and the others used Dräger PAC III [51,52]. In addition to CO, Wong et al. also measured CO_2_ simultaneously using TSI Q-Trak. Three measured HRV with a portable electrocardiograph (ECG) recorder or a smartwatch, while the other measured respiratory function using a portable mechanical volumetric spirometer. Focusing on daily life or traffic-related CO exposure scenarios, the studies recruited 7–44 healthy adults and collected personal environmental exposure and health data for 1–6 days. With high-resolution CO and HRV data, these studies were able to identify the lagged effects of CO exposure on HRV [29,51].

- Volatile Organic Compounds (VOCs)

Increased generalized volatile organic compounds (VOCs) include a large compound of organic pollutants, e.g., formaldehyde, benzene, toluene, xylene, phthalates, and polycyclic aromatic hydrocarbons (PAHs). TVOC often refers to the total VOCs measured from a sample using a TVOC sensor. VOC exposure has been associated with many adverse health effects, such as sick-building syndromes, asthma, blood dyscrasias, nasopharyngeal cancer, and leukemia [63]. The IHME estimates indicate that occupational exposure to formaldehyde and benzene led to about 3000 deaths and 136,000 DALYs worldwide in 2019, mainly from nasopharyngeal cancer and leukemia [1]. 

We found two studies examining the physiological effects of VOC exposure using portable sensors, as summarized in Table 2. While both studies measured TVOC via portable sensors, one used a commercial device that contained a photoionization detector [54], and the other used a self-made portable device comprising an electrochemical sensor [53]. As for physiological measurements, the former measured respiratory function with a portable spirometer supervised by staff, whereas the latter measured the resting metabolic rate with a portable indirect calorimeter. Moreover, both studies focused on the occupational population: 17 office workers in one study and 393 kitchen workers in the other. In addition, both studies examined the short-term effects, with 2 h exposure and measurements for each subject. No obvious correlation between VOC exposure and RMR was found. However, exposure to toxic air pollutants in gas-fueled kitchens led to worse lung functions and a higher prevalence of respiratory symptoms.

#### 3.3.2. Particles

Based on aerodynamic diameter sizes, ambient particles can be classified into ultrafine particles (UFPs or PM_0.1_; no larger than 0.1 μm), fine particles (PM_2.5_; no larger than 2.5 μm), inhalable particles (PM_10_; no larger than 10 μm), and total suspended particles (TSP; no larger than 100 μm). In addition, some other common pollutants are related to particles, including black carbon (BC) and particle-bounded PAHs (p-PAHs). Particle exposure has been associated with various human diseases, including cardiovascular diseases, lung cancers, diabetes, Alzheimer’s, depression [7], and anxiety [3,4,5,6,8]. The IHME estimates indicate that particle pollution resulted in about 6.5 million deaths and 209.6 million DALYs worldwide in 2019, representing the second largest risk factor for the global burden of diseases [1].

We found 16 eligible studies on particles, representing 67% of all of the included studies. The general information on exposure and health measurements, as well as study subjects, is shown in Table 3. The studies involved measurements of PM_2.5_ (16 studies), PM_10_ (5), PM_1_ (4), UFPs (3), PM_7_ (1), BC (3), and p-PAHs (1). While the particles were measured via portable devices in all studies, most used lab-grade devices (e.g., CPC and DiSCmini for UFPs, SidePak and DustTrak for PM_2.5_, and AE51 for BC) rather than lost-cost sensors. Only four studies utilized low-cost particle sensors, such as Dylos devices [64] and self-made boxes with PlanTower sensors [65,66,67]. Regarding health measurements, 13 studies measured HR or HRV, and 4 measured respiratory function (1 involved HR and respiratory functions). Despite varying makes and models, these studies used a Holter or ECG monitor to measure the HR and HRV, and a spirometer was used to measure the respiratory function. While most studies focused on healthy adults, Tang et al. (2007) recruited asthmatic children, and Arvind et al. [65] also recruited asthmatic subjects. Additionally, Xing et al. [68] examined hypertensive patients. Among the studies, the participants ranged from 7–282, and the monitoring periods ranged from hours to six days. As a spirometer is not a real-time device, the studies assessing participants’ respiratory function collected non-continuous health data. With high-resolution PM and HRV data, some studies were able to identify the lagged effects of PM exposure on HRV. For instance, Tsou et al. [66] found that short-term exposure to PM_2.5_ showed 6–18 h lag effects on overweight people’s HRV. In addition, Lee et al. [69] revealed that PM_2.5_ and BC exposure showed lagged effects on obese people’s HRV and HR at least within 3 h.

#### 3.3.3. Heavy Metals

Metals, especially heavy metals such as lead, cadmium, chromium, and arsenic pose a significant potential threat to human health in occupational and life situations. Heavy metals in the environment can enter and accumulate in the body through various pathways, including the respiratory, digestive, and dermal systems. Both high acute and chronic concentrations have been demonstrated as significant health risks [77,78]. The IHME estimates indicate that occupational exposure to arsenic, cadmium, nickel, and beryllium led to about 20,000 deaths and 560,000 DALYs worldwide in 2019, mainly from tracheal, bronchus, and lung cancer [1].

Over the last decade, environmental arsenic exposure has been a significant global public health concern, especially in drinking water. Nafees et al. conducted a comparative cross-sectional study to examine the associations between chronic arsenic exposure through drinking groundwater and a decrease in lung function among 200 subjects in total [76]. A portable kit was used to test the arsenic in water samples, and lung function was measured using a portable spirometer.

## 4. Discussion

Among the 16,751 papers identified in the initial search, most measured environmental factors or measured health responses with conventional methods, e.g., questionnaires [46,79], clinical visits [47,80], and biochemical analysis [81,82], rather than portable sensors. Overall, these studies using the above non-sensor methods consume vast amounts of human resources, materials, and time, thus limiting the scale of the study sample size and period. However, the included 24 studies examining environmental exposure and associated health effects via portable sensors/devices are generally also short-term and small-sample-based. Excluding the studies with one-time measurements, most included studies have a sample size of fewer than 100 participants and a study period of less than a week. It indicates that applying portable sensors to extensive human subject studies still faces substantial challenges. 

One of the main challenges is environmental sensors. Although the environmental monitors used in the included studies were relatively portable/mobile, they mostly added significant burdens to the participants due to their size and weight, preventing long-term personal exposure monitoring. The dimensions and weights of some portable environmental sensors/instruments for noise and chemical factors are summarized in Table S3. The listed sensors/instruments (e.g., Casella sound meter, 2B Tech. NO_x_ monitor, RAE TVOC monitor, TSI aerosol monitor) are generally in decimeters and kilograms. For instance, Tang et al. integrated several environmental sensors (UFPs, PM_2.5_, BC, p-PAHs) into a 6.6 kg system [29], challenging monitoring long-term personal exposure. In addition, the costs are relatively high, mostly in thousands of US dollars (USD). In contrast, the sensor for temperature monitoring (Thermochron iButton used in Runkle et al. [27]) was suitable for personal monitoring due to the small size (17 mm in diameter and 6 mm in thickness) and reasonable cost (~USD 50). On the other hand, the existing sensor-based studies mainly focus on chemical factors (NO_x_, CO_x_, TVOC, PM), with a few on physical factors (noise, temperature) and almost none on biological factors. However, many other environmental factors can affect human health, such as electromagnetic [83] and UV radiation [84] among physical factors; ozone, formaldehyde, benzene, and other VOCs among chemical factors; and pollen [85], viruses, bacteria, and fungi among biological factors. Almost no studies have investigated the above factors via sensors. Notably, low-cost, portable environmental sensors have been increasingly commercialized in recent years yet limited to light-scattering particle counters and electrochemical/semiconductor sensors for some gaseous components (NO_x_, CO_x_, ozone, SO_2_, TVOCs) [30,36]. For example, Mallire et al. measured ozone, TVOC, temperature, humidity, and activity levels with a 64 g wristband wearable device [86]. Moreover, studies on metal oxide (MOX) semiconductor gas sensors have drawn much attention due to their low costs, trim sizes, and reasonable life span. Some studies have developed prototypes of MOX-based gas sensors to measure a single VOC or multiple VOC components with sensor arrays [87,88,89]. In addition, sensor-based methods have been recently developed for per- and polyfluoroalkyl substances (PFAS) detection, such as small molecule complexation and assay-based methods, nanoparticle-based methods, molecularly imprinted polymer (MIP)-based methods, optical fiber-based method, and immunosensor-based methods [90]. Additionally, a few recent studies reviewed sensor-based methods for food contaminant detection, including pesticides, antibiotics, heavy metal ions, phenolic compounds, and nitrites [91,92,93]. Such sensors can be valuable in sensor-based environmental health research if commercialized. However, many of the above electrochemical and MOX sensors are still in the lab-based process. Thus, commercializing these sensors needs more work and cooperation from various parties. Developing multi-functional, small-size, and highly portable environmental sensors is of great value for future environmental monitoring research. 

Another challenge is health sensors. Among the 16,751 initial records, many studies measured health responses via conventional methods (e.g., questionnaires, personal reports, and clinical visits). The portable health sensors in the 24 eligible studies mainly include a smartwatch for HR and activity level monitoring, a Holter and ECG recorder for HRV monitoring, a BP monitor for BP measurement, and a spirometer for lung function measurements. The BP monitor and spirometer used in these studies were not real-time instruments—the measurements were performed manually. Currently, portable/wearable sensors for continuous health indicators are very limited. In addition to smartwatches (HR, physical activity, sleep quality, blood oxygen saturation), Holters (HRV), ECG recorders (HRV), and wrist BP monitors (BP), some companies (e.g., Omron and Huawei) have launched their smartwatch BP edition. However, these BP smartwatches cannot provide continuous BP monitoring as the user needs to trigger the measurement following a standard operating procedure. With advances in sensor technology, new portable sensors such as EEG signal monitoring [94], blood glucose monitoring [95], and electronic skin [38] are emerging. Commercial blood glucose sensing products have been increasingly popular, e.g., continuous glucose monitoring (CGM) products from Dexcom, Eversense, and FreeStyle Libre. However, the commercialization of flexible sensors needs more work on the sensor stability over long-term operations or the bending influence on sensing performance [96]. Future studies utilizing such emerging sensors can advance our understanding of the health effects of environmental exposure. 

**Figure 3:**
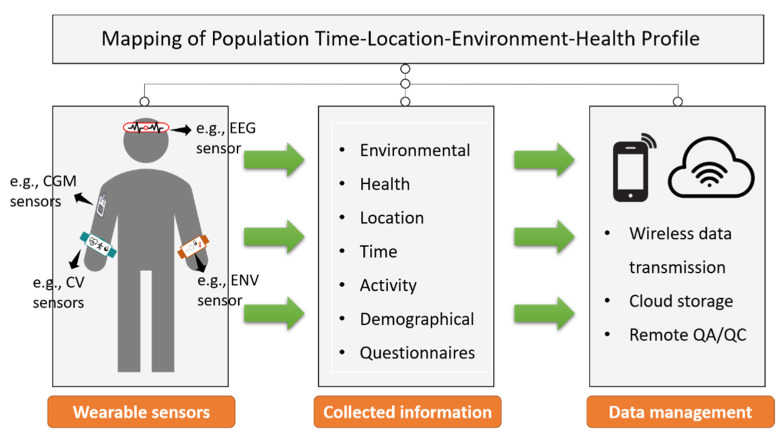
Illustration of human subject study framework using wearable environmental and health sensors. Abbreviations: EEG = electroencephalograph; CGM = continuous glucose monitoring; CV = cardiovascular; ENV = environment; QA/QC = quality assurance/ quality control.

Figure 3 illustrates a framework of human subject studies using environmental and health sensors. Wearable sensors placed on fingers, wrists, arms, necks, and heads are promising for personal exposure and health monitoring. Ideally, in a well-designed wearable sensor-based human subject study, various environmental and health-related information should be continuously monitored and collected to examine the health effects of environmental exposure. Information on time, location, demographic characteristics, and personal conditions should also be constantly collected to enable high spatial–temporal and stratified analysis. Measured data can be wirelessly transmitted to cloud servers and stored therein. Data quality can be assessed and controlled remotely through an algorithm applied to the servers. In addition, electronic questionnaires can be distributed to the study participants through the servers, avoiding the hassle of point-to-point distribution. Accordingly, with the sensors deployed (e.g., wearable environment/electroencephalograph/cardiovascular/continuous glucose monitoring sensors) and data collected, the population time–location–environment–health profiles can be mapped in detail, facilitating the analysis on various spatial and temporal scales. In addition, the real-time measurements of multiple factors and outcomes enable a comprehensive univariate and multivariate analysis.

We identify several aspects that can be investigated regarding population environmental health studies based on wearable smart sensors. First, a low-cost, small and light wearable device detecting multiple environmental factors or health indicators is needed. Ideally, one wearable device measures all environmental factors and health indicators. However, such devices can be enormous and thus unsuitable for wearable measurements. Therefore, multiple separate wearable devices can be more practical. Based on the literature and market investigations, small and tiny wearable devices for PM_2.5_, ozone, formaldehyde, some other VOCs, temperature, relative humidity, illuminance, HR, HRV, stress level, blood oxygen, blood glucose, and electroencephalograph (EEG) are available and valid. In addition to environmental and health sensors, location, physical activity, and sleep monitoring information are helpful and accessible. Wireless data transmission and battery life are also important. To make such devices low-cost, utilizing low-cost sensors, especially electrochemical sensors, is essential. Second, a quality assessment/control (QA/QC) method for such a study with large amounts of wearable sensors is necessary. The details about the sensor parameters (e.g., sensitivity, selectivity, and operational range) were not mentioned in studies included in the present analysis. However, the validation of some low-cost sensors, especially for particle sensors, in a controlled environment has been investigated well elsewhere [30,97,98,99,100,101]. Nevertheless, the validation of noise, light, temperature, and VOC sensors still needs more work. In addition, little is known about sensor validation during mobile/personal monitoring. Due to the relatively large variance of low-cost sensors, effective calibration techniques are required, and calibration must be done at intervals [102]. Several studies involving the calibration of mobile sensors via a rendezvous method [103,104] may be a direction for massive sensors. Moreover, validating the collected data also needs to be examined due to the uncertainties of whether the subject is wearing and using the device correctly [105]. Third, many wearable sensors developed in the last five years have not been used in population environmental health studies much. It can be an excellent opportunity to utilize those sensors and set up a standard framework for such studies.

## 5. Conclusions

This paper reviewed the growing number of case studies utilizing portable sensors/instruments to measure environmental exposures and the associated health effects in human subject studies. With a thorough literature review, 24 eligible studies were included and synthesized. The results show that particles were the most considered environmental factor among all physical, chemical, and biological factors, followed by TVOC and CO. HR and HRV were the most considered health indicators among all cardiopulmonary outcomes, followed by respiratory function. The studies mostly had a sample size of fewer than 100 participants and a study period of less than a week due to the challenges in accessing low-cost, small, and light wearable sensors. Future studies may benefit from the following aspects: (1) building low-cost, small, and light wearable devices which can detect multiple environmental factors or health indicators by utilizing various sensors; (2) developing QA/QC methods specific to human subject studies that use massive sensors; and (3) commercializing and utilizing the wearable sensors which have been developed in last five years in such human subject studies. With the advances in sensor development, more low-cost, high-precision, and long-endurance wearable sensors can be used for large-scale environmental health studies. This review guides future sensor-based environmental health studies on project design and sensor selection.

## Figures and Tables

**Table 1:** Studies focusing on health effects of physical factor exposure.

Study	Location	Scenario	Period	Subject	Exposure Measurement	Health Measurement	Number of Records	Main Findings
Nserat et al. [41]	Jordan	Industrial plants	2017	191 male workers	Noise level: Casella sound level meter CEL-450A, ~USD 4000	Blood pressure (BP): KaWe Mastermed A2 Aneroid BP Monitor, ~USD 43	One time for each subject	Exposure to a high level of noise was associated with elevated blood pressure.
Cole-Hunter et al. [42]	Spain	Traffic	2011	28 healthy non-smoking adults	Noise level (LAeq): CESVA sound level meter SC160	Heart rate (HR), heart rate variability (HRV): Gem-Med Holter monitor CardioLight	8 h for each subject	Not presented.
Runkle et al. [27]	USA	Occupational	2016	35 outdoors workers	Ambient temperature: Thermochron iButton DS 1921G, ~USD 50	HR: Garmin vivoActive HR watches, ~USD 1500	5 days for each subject	The association between increasing temperature and heat strain was nonlinear and exhibited a U-shaped relationship.
Sugg et al. [43]	USA	Occupational	2018	54 outdoors workers	Ambient temperature, solar radiation intensity: Thermochron iButton DS 1921G, ~USD 50	HR: Garmin vivoActive HR watches, ~USD 1500	1 week for each subject	A weak significant relationship was observed between personal ambient temperatures and weather station measurements.
Basu and Samet [44]	USA	Daily routine	2000	42 elderly residents	Ambient temperature: unknown temperature sensor probes	HR, body temperature: unknown polar chest strap, temperature sensor probes, mercury detectors	48 h for each subject	Body temperature was not associated with ground station temperature.

**Table 2:** Studies focusing on health effects of gaseous pollutant exposure.

Study	Location	Scenario	Period	Subject	Exposure Measurement	Health Measurement	Number of Records	Main Findings
Matt et al. [50]	Spain	Traffic	2013–2014	30 healthy adults	NO_x_: 2B Tech. Model 410 Nitric Oxide Monitor, ~USD 8000	Respiratory function: Ndd Medical EasyOne spirometer, ~USD 1900	8 h for each subject	Associations between NO_x_ exposure and respiratory measures were modified by participants’ physical activity levels.
Tang et al. [29]	China	Daily life	2012–2013	7 healthy older people	CO: TSI Q-TRAK model 7575, ~USD 4300	HRV: MSI ECG recorder and analyzer model E3-8010	144 h for each subject	Exposure to CO had a lagged effect of 0–7 h on HRV for elders.
Tang et al. [51]	China	Traffic	2009–2010	20 college students	CO: Dräger PAC III CO detection instrument, ~USD 900	HRV: MSI ECG recorder and analyzer model E3-8010	48 h for each subject	Exposure to CO had a > 4 h lagged effect on HRV for young people.
Saadi et al. [52]	Israel	Daily life	Not mentioned	44 healthy women	CO: Dräger PAC III CO detection instrument, ~USD 900	HRV: Polar 810i monitor	48 h for each subject	Short-term exposure to CO below 7 ppm was related to declined HRV.
Deng et al. [53]	USA	Working and resting	2016	17 workers	TVOC: Self-made portable wireless VOC monitoring device	Individual resting metabolic rate (RMR): Breezing Indirect Calorimeter, ~USD 550	2 h for each subject	No obvious correlation between VOCs exposure and RMR was found.
Wong et al. [54]	China	Chinese restaurant kitchens	Not mentioned	393 kitchen workers	CO, CO_2_: TSI Q-Trak Model 8554, TVOC: RAE Systems PGM-7240, ~USD 1200	Respiratory function: Vitalograph 2160	2 h for each subject	Exposure to toxic air pollutants in kitchens led to worse lung functions and higher prevalence of respiratory symptoms.

**Table 3:** Studies focusing on health effects of particle and heavy metal exposure.

Study	Location	Scenario	Period	Subject	Exposure Measurement	Health Measurement	Number of Records	Main Findings
Lee et al. [64]	Korea	Daily life	2018–2019	22 healthy adults	PM_2.5_: Dylos DC1700	BP: IEM Mobil-O Graph Ambulatory BP monitor HR and HRV: Aria Del Mar Reynolds Medical ECG monitor	24 h for each subject	Short-term exposure to PM_2.5_ was associated with decreased HRV.
Tang et al. [29]	China	Daily life	2012–2013	7 healthy older adults	UFPs: DiSCmini PM_2.5_ and PM_10_: Grimm PAS Model 1.109 BC: MicroAeth model AE51 p-PAHs: EcoChem Photoelectric sensor PAS2000CE	HRV: MSI ECG Model E3-8010	144 h for each subject	Different pollutants showed different lagged effects on HRV.
Tsou et al. [66]	China	Daily life	2018–2019	35 healthy adults	PM_1_ and PM_2.5_: Self-made box with PlanTower PMS sensor	HRV: RootiRx	48 h for each subject	Short-term exposure to PM_2.5_ had 6–18 h lagged effects on overweight people’s HRV.
Cole-Hunter et al. [42]	Spain	Traffic	2011	28 healthy non-smoking adults	UFPs: TSI CPC Model 3007 PM_2.5_: TSI DusTrak Model 8532 BC: MicroAeth model AE51	HR and HRV: Gem-Med Holter monitor CardioLight	8 h for each subject	Exposure to TRAP shows a rapid but nonlinear impact on HRV in healthy adults.
He et al. [70]	USA	Daily life	2007–2009	106 healthy non-smoking elders	PM_2.5_: Thermo Scientific Personal DataRam pDR model 1200	HR: Mortara 12-lead HScribe Holter System	24 h for each subject	PM_2.5_ exposure was related to HRV, with the largest effects occurring about 4–6 h lagged.
Lee et al. [69]	USA	Daily life	2004	21 healthy adults	PM_2.5_: TSI SidePak AM510	HR and HRV: Raytel Cardiac Services ECG Holter	48 h for each subject	Short-term exposure to PM_2.5_ showed a lag effect on people’s HRV up to 2.5 h.
Li et al. [71]	China	Daily life	2017–2018	97 young adults	PM_2.5_: RTI MicroPEM BC: MicroAeth model AE51	HR and HRV: DM Software Inc. 12-channel Holter recorder MGY-H12	24 h for each subject	PM_2.5_/BC exposure showed lag effects on obese people’s HRV and HR at least within 3 h.
Lung et al. [67]	China	Daily life	Not mentioned	36 healthy non-smoking adults	PM_2.5_: Self-made box with PlanTower PMS sensor	HRV: RootiRx	48–96 h for each subject	Short-term exposure to low-level PM_2.5_ (<10 µg/m^3^) was related to HRV.
Magari et al. [72]	USA	Industrial plants	Not mentioned	40 male workers	PM_2.5_: TSI DustTrak 8534	HRV: Dynacord 3-channel device 423	up to 24 h for each subject	Occupational and environmental PM_2.5_ exposure within minutes to hours was related to HRV.
Langrish et al. [73]	China	Daily life	2008	15 healthy non-smoking volunteers	PM_2.5_: Thermo Scientific DataRAM monitor pDR-1500	HRV: Spacelabs Holter monitor Lifecard	24 h for each subject	Wearing a mask for 2 h tended to eliminate the adverse effects of air pollution on blood pressure and HRV.
Matt et al. [50]	Spain	Traffic	2013–2014	30 healthy non-smoking adults	UFPs: TSI CPC 3007 PM_2.5_ and PM_10_: TSI DustTrack 8534	HR: Gem-Med Holter monitor CardioLight Respiratory function: Ndd Medical EasyOne spirometer	8 h for each subject	Associations between various pollutant exposures and respiratory measures were modified by participants’ physical activity levels.
Tang et al. [51]	China	Traffic	2009–2010	20 healthy college students	PM_1_, PM_2.5,_ and PM_10_: GRIMM PAS Model 1.108	HRV: MSI ECG Model E3-8010	48 h for each subject	Exposure to PM_2.5_–10, among all size-fractional particles, led to the largest variations in HRV.
Tang et al. [74]	China	Daily life	2003–2005	30 children with asthma	PM_1_, PM_2.5,_ and PM_10_: GRIMM PAS Model 1.108	Peak expiratory flow rate (PEFR): Microlife Electronic PEFR monitor PF-100	14 h for each subject	PM exposure showed lagged and cumulative effects on the decrements in morning PEFR.
Arvind et al. [65]	Greece	Daily life	Not mentioned	44 asthmatic subjects	PM_2.5_: Airpseck sensor (self-made)	Respiratory rate: Respeck sensor (self-made)	48 h for each subject	Short-term exposure to PM_2.5_ showed lagged effects on respiratory rates of asthmatic adolescents
Xing et al. [68]	China	Daily life	2017–2019	282 hypertension patients	PM_2.5_: RTI MicroPEM and TSI SidePak AM520	HRV: 12-lead Holter device. JincoMed	3 days for each subject	Short-term exposure to PM_2.5_ was related to HRV; BP control and ARB treatment alleviated the adverse effects.
Nyhan et al. [75]	Ireland	Traffic	Not mentioned	32 young, healthy subjects	PM_1_, PM_2.5_, PM_7_, PM_10_, and TSP: Met One Aerocet 531	HRV: CamNtech Actiheart units	8–10 h for each subject	Short-term exposure to PM_2.5_ was related to HRV decline for commuters.
Nafees et al. [76]	Pakistan	Drinking groundwater	2009	100 subjects ≥15 yrs	Water Arsenic: Industrial Test Systems, Inc. Arsenic Quick Kit	Lung function: Vitalograph New Alpha 6000 spirometer	One time for each subject	Chronic exposure to arsenic in drinking groundwater was associated with a decrement in lung function.

## Data Availability

All data needed to evaluate the conclusions in the paper are present in the paper and/or the Supplementary Materials. Additional data related to this paper are available upon request.

## Supplementary Materials

The following supporting information can be downloaded at: https://www.mdpi.com/article/10.3390/bios12121131/s1, Table S1: Literature search strategy; Table S2: Summary of the included studies; Table S3: Characteristics of some portable instruments for environmental measurements; Figure S1: Illustration of the typical sensors and the wearable positions in the included studies.
