# Supplementary material for: Wearable Sensor-Based Monitoring of Environmental Exposures and the Associated Health Effects: A Review
Source: Biosensors (Basel). 2022 Dec 6;12(12):1131. doi: 10.3390/bios12121131 (PMC9775571; doi:10.3390/bios12121131)
Supplement: Supplementary file 1 [file biosensors-12-01131-s001.zip › biosensors-2045186-supplementary.pdf]

Wearable Sensor-Based Monitoring of Environmental Exposures and the Associated Health Effects: A Review

Xueer Lin [1], Jiaying Luo [1], Minyan Liao [1], Yalan Su [1], Mo Lv [1], Qing Li [2], Shenglan Xiao [3,*] and Jianbang Xiang [1,4,*]

[1] School of Public Health (Shenzhen), Sun Yat-sen University, Shenzhen 518107, China
[2] College of Medicine and Biological Information Engineering, Northeastern University, Shenyang 110819, China
[3] School of Public Health (Shenzhen), Shenzhen Campus of Sun Yat-sen University, Shenzhen 518107, China
[4] State Environmental Protection Key Laboratory of Sources and Control of Air Pollution Complex, Beijing 100084, China
* Correspondence: xiangjb@mail.sysu.edu.cn (J.X.); xiaoshlan3@mail.sysu.edu.cn (S.X.)

The supplementary materials contain three tables and one figure.

Table S1. Literature search strategy.

Database	No.	Query	Records
Web of Science	1	TS= (environmental factor*) AND TS = (using wearable*) AND TS = (health effect*)	47
	2	TS=(environmental (factor* OR exposure)) AND TS=(using (wearable OR mobile OR portable)) AND TS=(health effect*)	1828
	3	TS=(nois*(factor* OR exposure)) AND TS=(using (wearable OR mobile OR portable)) AND TS=(health effect*)	192
	4	TS=(heat (factor* OR exposure)) AND TS=(using (wearable OR mobile OR portable)) AND TS=(health effect*)	258
	5	TS=(temperature (factor* OR exposure)) AND TS=(using (wearable OR mobile OR portable)) AND TS=(health effect*)	470
	6	TS=(humid* (factor* OR exposure)) AND TS=(using (wearable OR mobile OR portable)) AND TS=(health effect*)	116
	7	TS=(radiat* (factor* OR exposure)) AND TS=(using (wearable OR mobile OR portable)) AND TS=(health effect*)	1808
	8	TS=(UV*(factor* OR exposure)) AND TS=(using (wearable OR mobile OR portable)) AND TS=(health effect*)	106
	9	TS=(electromagnetic *(factor* OR exposure)) AND TS=(using (wearable OR mobile OR portable)) AND TS=(health effect*)	1270
	10	TS=(ozone (factor* OR exposure)) AND TS=(using (wearable OR mobile OR portable)) AND TS=(health effect*)	97
	11	TS=((nitrogen* OR NO) (factor* OR exposure)) AND TS=(using (wearable OR mobile OR portable)) AND TS=(health effect*)	1769
	12	TS=((NH3 OR ammonia) (factor* OR exposure)) AND TS=(using (wearable OR mobile OR portable)) AND TS=(health effect*)	30
	13	TS=(chemical* (factor* OR exposure)) AND TS=(using (wearable OR mobile OR portable)) AND TS=(health effect*)	799
	14	TS=(organic* (factor* OR exposure)) AND TS=(using (wearable OR mobile OR portable)) AND TS=(health effect*)	625
	15	TS=(inorganic* (factor* OR exposure)) AND TS=(using (wearable OR mobile OR portable)) AND TS=(health effect*)	36
	16	TS=(formaldehyde (factor* OR exposure)) AND TS=(using (wearable OR mobile OR portable)) AND TS=(health effect*)	36
	17	TS=((VOC* OR TVOC*)(factor* OR exposure)) AND TS=(using (wearable OR mobile OR portable)) AND TS=(health effect*)	68
	18	TS=((partic* OR PM) (factor* OR exposure)) AND TS=(using (wearable OR mobile OR portable)) AND TS=(health effect*)	3145
	19	TS=(virus (factor* OR infect*)) AND TS=(using (wearable OR mobile OR portable)) AND TS=(health effect*)	1181
	20	TS=(((bacteria OR virus OR fungi) exposure) AND (using(wearable OR mobile OR portable))AND (health effect*))	211
PubMed	21	(environment*) AND (using wearable*) AND (health effect*)	24
	22	(environment*) AND (using wearable OR mobile OR portable)) AND (health effect*)	224
	23	(noise) AND (using wearable OR mobile OR portable) AND (health effect*)	11
	24	(heat) AND (using wearable OR mobile OR portable) AND (health effect*)	7
	25	(temperature) AND (using wearable OR mobile OR portable) AND (health effect*)	23
	26	(humid*) AND (using wearable OR mobile OR portable) AND (health effect*)	8
	27	(radiat*) AND (using wearable OR mobile OR portable) AND (health effect*)	71

Database	No.	Query	Records
	28	(UV*) AND (using wearable OR mobile OR portable) AND (health effect*)	8
	29	(electromagnetic) AND (using wearable OR mobile OR portable) AND (health effect*)	71
	30	(ozone) AND (using wearable OR mobile OR portable) AND (health effect*)	0
	31	(nitrogen* OR NO) AND (using wearable OR mobile OR portable) AND (health effect*)	5
	32	(NH3 OR ammonia) AND (using wearable OR mobile OR portable) AND (health effect*)	1
	33	(chemical) AND (using wearable OR mobile OR portable) AND (health effect*)	35
	34	(organic*) AND (using wearable OR mobile OR portable) AND (health effect*)	4
	35	(inorganic*) AND (using wearable OR mobile OR portable) AND (health effect*)	0
	36	(formaldehyde) AND (using wearable OR mobile OR portable) AND (health effect*)	0
	37	(VOC* OR TVOC*) AND (using wearable OR mobile OR portable) AND (health effect*)	1
	38	(partic* OR PM)) AND (using wearable OR mobile OR portable) AND (health effect*)	1839
	39	(virus factor* OR infect*) AND (using wearable OR mobile OR portable) AND (health effect*)	228
	40	(bacteria OR virus OR fungi) AND (using wearable OR mobile OR portable) AND (health effect*)	99

Table S2. Summary of the included studies.

Study	Title	Year	Location	Media	Environmental Factor
[1]	Blood Pressure of Jordanian Workers Chronically Exposed to Noise in Industrial Plants	2017	Jordan	Air	Noise
[2]	Evaluation of wearable sensors for physiologic monitoring of individually experienced temperatures in outdoor workers in southeastern US	2019	USA	Air	Temperature
[3]	Estimating personal ambient temperature in moderately cold environments for occupationally exposed populations	2019	USA	Air	Temperature
[4]	An exposure assessment study of ambient heat exposure in an elderly population in Baltimore, Maryland	2002	USA	Air	Temperature
[5]	Lung function decrement with arsenic exposure to drinking groundwater along River Indus: a comparative cross-sectional study	2011	Pakistan	Water	Metal
[6]	Identifying causal relationships in time-series data from a pair of wearable sensors	2021	UK	Air	PM
[7]	Effect of short-term exposure to particulate air pollution on heart rate variability in normal-weight and obese adults	2021	China	Air	PM
[8]	A community-based study on associations between PM2.5 and PM1 exposure and heart rate variability using wearable low-cost sensing devices	2021	China	Air	PM
[9]	Declines in heart rate variability associated with short-term PM2.5 exposure were modified by blood pressure control and treatment: A multi-city panel study in China	2021	China	Air	PM
[10]	Personal exposure to fine particulate air pollutants impacts blood pressure and heart rate variability	2020	USA	Air	PM
[11]	Panel study using novel sensing devices to assess associations of PM(2.5)with heart rate variability and exposure sources	2020	China	Air	PM
[12]	Effects of Personal Exposures to Micro- and Nano-Particulate Matter, Black Carbon, Particle-Bound Polycyclic Aromatic Hydrocarbons, and Carbon Monoxide on Heart Rate Variability in a Panel of Healthy Older Subjects	2019	China	Air	PM, CO
[13]	Impact of traffic-related air pollution on acute changes in cardiac autonomic modulation during rest and physical activity: a cross-over study	2016	Spain	Air	PM, Noise
[14]	Effects of Personal Exposure to Ambient Fine Particulate Matter on Acute Change in Nocturnal Heart Rate Variability in Subjects Without Overt Heart Disease	2016	USA	Air	PM

Study	Title	Year	Location	Media	Environmental Factor
[15]	Comparison of particulate matter dose and acute heart rate variability response in cyclists, pedestrians, bus and train passengers	2014	Ireland	Air	PM
[16]	Individual-level PM2.5 exposure and the time course of impaired heart rate variability: the APACR Study	2011	USA	Air	PM
[17]	Beneficial cardiovascular effects of reducing exposure to particulate air pollution with a simple facemask	2009	China	Air	PM
[18]	Effects of personal particulate matter on peak expiratory flow rate of asthmatic children	2007	China	Air	PM
[19]	Association of heart rate variability with occupational and environmental exposure to particulate air pollution	2001	USA	Air	PM
[20]	Impacts of In-Cabin Exposure to Size-Fractionated Particulate Matters and Carbon Monoxide on Changes in Heart Rate Variability for Healthy Public Transit Commuters	2019	China	Air	PM, CO
[21]	Acute respiratory response to traffic-related air pollution during physical activity performance	2016	Spain	Air	PM, NOx
[22]	The Relationship between City Size and Carbon Monoxide (CO) Concentration and Their Effect on Heart Rate Variability (HRV)	2021	Israel	Air	CO
[23]	Tracking Personal Health-Environment Interaction with Novel Mobile Sensing Devices	2018	USA	Air	TVOCs
[24]	Respiratory health and lung function in Chinese restaurant kitchen workers	2011	China	Air	CO, CO2, TVOCs

Table S3. Characteristics of some portable instruments for environmental measurements.

**Environmental factor**	**Instrument**	**Dimensions (mm)**	**Weight (kg)**	**Price (US dollars)**	**Link**
Noise	Casella sound level meter CEL-450A	340 x 100 x 40	0.55	~4000	https://www.jjstech.com/cel-450-c2-k1.html
NOx	2B Tech. Model 410 Nitric Oxide Monitor	130 x 210 x 340	3.7	~8000	https://twobtech.com/docs/manuals/model_410_revE.pdf
TVOCs	RAE Systems PGM-7240	255 x 76 x 64	0.738	~4000	https://www.frontline-safety.co.uk/ppbrae-3000-model-pgm-7340-monitor-accessories-kit
PM2.5	TSI DustTrak 8534	125 x 121 x 316	1.3	~5000	https://norrscope.com/product/tsi-8534-dusttrak-drx/
UFPs	TSI CPC 3007	292 x 140 x 140	1.7	~5000	https://tsi.com/getmedia/a1852300-29bc-4a4b-acaa-4220bfd65334/3007_1930032-CPC-Spec-Sheet-US?ext=.pdf

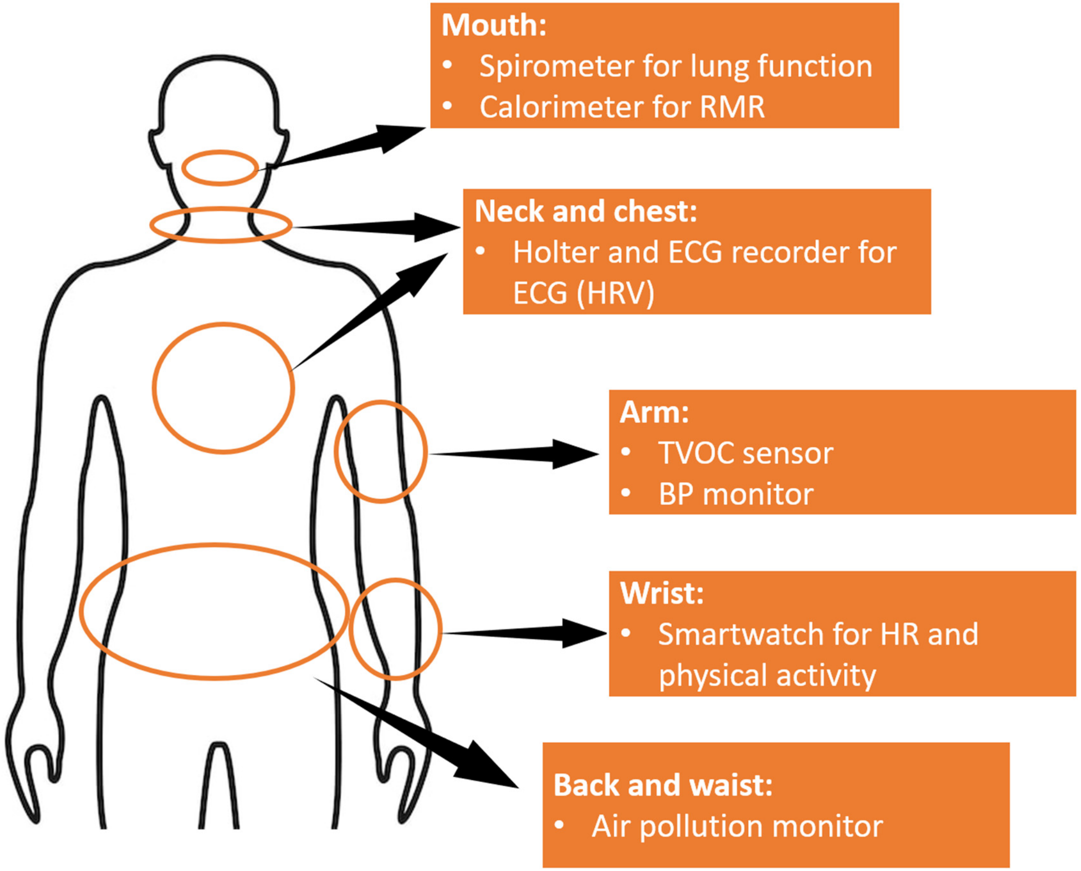

Figure S1. Illustration of the typical sensors and the wearable positions in the included studies.

References

1. Nserat, S.; Al-Musa, A.; Khader, Y.S.; Abu Slaih, A.; Iblan, I. Blood pressure of jordanian workers chronically exposed to noise in industrial plants. *International Journal of Occupational and Environmental Medicine* **2017**, *8*, 217-223. https://doi:10.15171/ijoem.2017.1134.
2. Runkle, J.D.; Cui, C.; Fuhrmann, C.; Stevens, S.; Del Pinal, J.; Sugg, M.M. Evaluation of wearable sensors for physiologic monitoring of individually experienced temperatures in outdoor workers in southeastern US. *Environ. Int.* **2019**, *129*, 229-238. https://doi:10.1016/j.envint.2019.05.026.
3. Sugg, M.M.; Stevens, S.; Runkle, J.D. Estimating personal ambient temperature in moderately cold environments for occupationally exposed populations. *Environ. Res.* **2019**, *173*, 497-507. https://doi:10.1016/j.envres.2019.03.066.
4. Basu, R.; Samet, J.M. An exposure assessment study of ambient heat exposure in an elderly population in Baltimore, Maryland. *Environ. Health Perspect.* **2002**, *110*, 1219-1224. https://doi:10.1289/ehp.021101219.
5. Nafees, A.A.; Kazi, A.; Fatmi, Z.; Irfan, M.; Ali, A.; Kayama, F. Lung function decrement with arsenic exposure to drinking groundwater along River Indus: a comparative cross-sectional study. *Environ. Geochem. Health* **2011**, *33*, 203-216. https://doi:10.1007/s10653-010-9333-7.
6. Arvind, D.K.; Maiya, S.; Sedeno, P.A. Identifying causal relationships in time-series data from a pair of wearable sensors. In Proceedings of the 2021 IEEE 17th International Conference on Wearable and Implantable Body Sensor Networks (BSN), Athens, Greece, 27–30 July 2021. https://doi.org/10.1109/bsn51625.2021.9507030.
7. Li, L.Y.; Hu, D.Y.; Zhang, W.L.; Cui, L.Y.; Jia, X.; Yang, D.; Liu, S.; Deng, F.R.; Liu, J.X.; Guo, X.B. Effect of short-term exposure to particulate air pollution on heart rate variability in normal-weight and obese adults. *Environ. Health* **2021**, *20*, 29. https://doi:10.1186/s12940-021-00707-0.
8. Tsou, M.C.M.; Lung, S.C.C.; Shen, Y.S.; Liu, C.H.; Hsieh, Y.H.; Chen, N.; Hwang, J.S. A community-based study on associations between PM2.5 and PM1 exposure and heart rate variability using wearable low-cost sensing devices. *Environ. Pollut.* **2021**, *277*, 116761. https://doi.org/10.1016/j.envpol.2021.116761.
9. Xing, X.L.; Liu, F.C.; Yang, X.L.; Liu, Q.; Wang, X.Y.; Lin, Z.N.; Huang, K.Y.; Cao, J.; Li, J.X.; Fan, M.; et al. Declines in heart rate variability associated with short-term PM2.5 exposure were modified by blood pressure control and treatment: A multi-city panel study in China*. *Environmental Pollution* **2021**, *287*, 117572. https://doi:10.1016/j.envpol.2021.117572.
10. Lee, D.H.; Kim, S.H.; Kang, S.H.; Kwon, O.K.; Park, J.J.; Yoon, C.H.; Cho, Y.S.; Heo, J.; Yi, S.M.; Youn, T.J.; et al. Personal exposure to fine particulate air pollutants impacts blood pressure and heart rate variability. *Sci. Rep.* **2020**, *10*, 16538. https://doi:10.1038/s41598-020-73205-x.
11. Lung, S.C.C.; Chen, N.; Hwang, J.S.; Hu, S.C.; Wang, W.C.V.; Wen, T.Y.J.; Liu, C.H. Panel study using novel sensing devices to assess associations of PM(2.5)with heart rate variability and exposure sources. *J. Expo. Sci. Environ. Epidemiol.* **2020**, *30*, 937-948. https://doi:10.1038/s41370-020-0254-y.
12. Tang, C.-S.; Chuang, K.-J.; Chang, T.-Y.; Chuang, H.-C.; Chen, L.-H.; Lung, S.-C.C.; Chang, L.-T. Effects of personal exposures to micro- and nano-particulate matter, black carbon, particle-bound polycyclic aromatic hydrocarbons, and carbon monoxide on heart rate variability in a panel of healthy older subjects. *Int. J. Env. Res. Public Health* **2019**, *16*, 4672. https://doi:10.3390/ijerph16234672.
13. Cole-Hunter, T.; Weichenthal, S.; Kubesch, N.; Foraster, M.; Carrasco-Turigas, G.; Bouso, L.; Martinez, D.; Westerdahl, D.; de Nazelle, A.; Nieuwenhuijsen, M. Impact of traffic-related air pollution on acute changes in cardiac autonomic modulation during rest and physical activity: A cross-over study. *J. Expo. Sci. Environ. Epidemiol.* **2016**, *26*, 133-140. https://doi:10.1038/jes.2015.66.
14. Lee, M.S.; Eum, K.D.; Rodrigues, E.G.; Magari, S.R.; Fang, S.C.; Modest, G.A.; Christiani, D.C. Effects of personal exposure to ambient fine particulate matter on acute change in

nocturnal heart rate variability in subjects without overt heart disease. *Am. J. Cardiol.* **2016**, *117*, 151-156. https://doi:10.1016/j.amjcard.2015.10.015.
15. Nyhan, M.; McNabola, A.; Misstear, B. Comparison of particulate matter dose and acute heart rate variability response in cyclists, pedestrians, bus and train passengers. *Sci. Total Environ.* **2014**, *468*, 821-831. https://doi:10.1016/j.scitotenv.2013.08.096.
16. He, F.; Shaffer, M.L.; Li, X.A.; Rodriguez-Colon, S.; Wolbrette, D.L.; Williams, R.; Cascio, W.E.; Liao, D.P. Individual-level PM2.5 exposure and the time course of impaired heart rate variability: the APACR Study. *J. Expo. Sci. Environ. Epidemiol.* **2011**, *21*, 65-73. https://doi:10.1038/jes.2010.21.
17. Langrish, J.P.; Mills, N.L.; Chan, J.K.K.; Leseman, D.; Aitken, R.J.; Fokkens, P.H.B.; Cassee, F.R.; Li, J.; Donaldson, K.; Newby, D.E.; et al. Beneficial cardiovascular effects of reducing exposure to particulate air pollution with a simple facemask. *Part. Fibre Toxicol.* **2009**, *6*, 8. https://doi:10.1186/1743-8977-6-8.
18. Tang, C.S.; Chang, L.T.; Lee, H.C.; Chan, C.C. Effects of personal particulate matter on peak expiratory flow rate of asthmatic children. *Sci. Total Environ.* **2007**, *382*, 43-51. https://doi:10.1016/j.scitotenv.2007.04.016.
19. Magari, S.R.; Hauser, R.; Schwartz, J.; Williams, P.L.; Smith, T.J.; Christiani, D.C. Association of heart rate variability with occupational and environmental exposure to particulate air pollution. *Circulation* **2001**, *104*, 986-991. https://doi:10.1161/hc3401.095038.
20. Tang, C.S.; Wu, T.Y.; Chuang, K.J.; Chang, T.Y.; Chuang, H.C.; Lung, S.C.C.; Chang, L.T. Impacts of in-cabin exposure to size-fractionated particulate matters and carbon monoxide on changes in heart rate variability for healthy public transit commuters. *Atmosphere* **2019**, *10*, 409. https://doi:10.3390/atmos10070409.
21. Matt, F.; Cole-Hunter, T.; Donaire-Gonzalez, D.; Kubesch, N.; Martinez, D.; Carrasco-Turigas, G.; Nieuwenhuijsen, M. Acute respiratory response to traffic-related air pollution during physical activity performance. *Environ. Int.* **2016**, *97*, 45-55. https://doi:10.1016/j.envint.2016.10.011.
22. Saadi, D.; Tirosh, E.; Schnell, I. The relationship between city size and carbon monoxide (CO) concentration and their effect on heart rate variability (HRV). *Int. J. Env. Res. Public Health* **2021**, *18*, 788. https://doi:10.3390/ijerph18020788.
23. Deng, Y.; Liu, N.Y.; Tsow, F.; Xian, X.J.; Krajmalnik-Brown, R.; Tao, N.J.; Forzani, E. Tracking personal health-environment interaction with novel mobile sensing devices. *Sensors* **2018**, *18*, 2670. https://doi:10.3390/s18082670.
24. Wong, T.W.; Wong, A.H.S.; Lee, F.S.C.; Qiu, H. Respiratory health and lung function in Chinese restaurant kitchen workers. *Occup. Environ. Med.* **2011**, *68*, 746-752. https://doi:10.1136/oem.2010.059378.
